# PD179 Navigating Health Crises In Emerging Economies: A Comprehensive Examination Of COVID-19’s Influence On Health Care Access And Resilience

**DOI:** 10.1017/S0266462324004045

**Published:** 2025-01-07

**Authors:** Birol Tibet, Ekin Begum Ozdemir, Yaren Erkut, Selin Okcun, Mustafa Kurnaz, Guvenc Kockaya

## Abstract

**Introduction:**

This research focuses on the impact of the COVID-19 pandemic on health care in emerging markets. It examines the pandemic’s effects on emergency surgeries, medical consultations, maternal care, primary health care services, and surgical interventions.

**Methods:**

This study focused on assessing the effect of COVID-19 on healthcare services in emerging markets from 2019 to 2021. It involved a literature review of academic articles, health reports, and government data, targeting the pandemic’s effect on healthcare access. Data were collected from official health records in Brazil, China, Ethiopia, Egypt, Poland, Qatar, Sub-Saharan Africa, Thailand, and Türkiye. The analysis focused on emergency surgeries, outpatient visits, hospital admissions, primary health care, prenatal and maternal care, clinic visits for cardiac implantable electronic devices (CIED), and general surgeries, aiming to understand changes in health care access during and after the pandemic.

**Results:**

Emergency surgeries in Ethiopia decreased by 77 percent, while in Egypt there was a 66.4 percent reduction in chest clinic visits. Outpatient and hospital admissions fell by 7 to 17 percent in Sub-Saharan Africa. In China, hospital, primary care, and inpatient visits declined by 33, 71, and 42 percent, respectively. In Qatar, physical healthcare visits fell by 36 percent, though virtual consultations increased notably. CIED visits in Poland fell by 26 percent in 2020. Thailand struggled with increased COVID-19 cases and deaths in 2021, whereas Brazil’s health care services were significantly reduced (42.6% reduction in screenings and 59.7% reduction in surgeries). In Türkiye, there was a 35 percent drop in hospital visits and a 15 percent drop in prescriptions, but with increased costs per visit (0.09%) and per prescription (42.3%).

**Conclusions:**

This study highlights the profound impact of COVID-19 on health care in emerging markets, showing significant disruptions in services like surgeries and outpatient visits. The pandemic emphasized the necessity of robust, adaptable healthcare systems and accelerated the shift to digital health services. These findings urge the strengthening of healthcare in emerging markets to prepare them for future global health challenges.

